# Ovarian follicle size or growth rate can both be determinants of ovulatory follicle selection in mice^†^

**DOI:** 10.1093/biolre/ioad134

**Published:** 2023-10-06

**Authors:** Sharon Richard, Yiran Zhou, Christine L Jasoni, Michael W Pankhurst

**Affiliations:** Department of Anatomy, School of Biomedical Sciences, University of Otago, Dunedin, New Zealand; Department of Anatomy, School of Biomedical Sciences, University of Otago, Dunedin, New Zealand; Department of Anatomy, School of Biomedical Sciences, University of Otago, Dunedin, New Zealand; Centre for Neuroendocrinology, School of Biomedical Sciences, University of Otago, Dunedin, New Zealand; Department of Anatomy, School of Biomedical Sciences, University of Otago, Dunedin, New Zealand

**Keywords:** polyovulatory, Graafian follicle, folliculogenesis, dominant follicle, proliferation model

## Abstract

The endocrinology regulating ovulation of the desired number of oocytes in the ovarian cycle is well described, particularly in mono-ovulatory species. Less is known about the characteristics that make one follicle suitable for ovulation while most other follicles die by atresia. Bromodeoxyuridine (BrdU) injection was used to characterize granulosa cell proliferation rates in developing ovarian follicles in the estrous cycle of mice. This methodology allowed identification of follicle diameters of secondary (80–130 μm), follicle-stimulating hormone (FSH)-sensitive (130–170 μm), FSH-dependent (170–350 μm), and preovulatory (>350 μm) follicles. Few preovulatory-sized follicles were present in the ovaries of mice at estrus, the beginning of the cycle. Progressive increases were seen at metestrus and diestrus, when full accumulation of the preovulatory cohort (~10 follicles) occurred. BrdU pulse-chase studies determined granulosa cell proliferation rates in the 24–48 h before the follicle reached the preovulatory stage. This showed that slow-growing follicles were not able to survive to the preovulatory stage. Mathematical modeling of follicle growth rates determined that the largest follicles at the beginning of the cycle had the greatest chance of becoming preovulatory. However, smaller follicles could enter the preovulatory follicle pool if low numbers of large antral follicles were present at the beginning of the cycle. In this instance, rapidly growing follicles had a clear selection advantage. The developing follicle pool displays heterogeneity in granulosa cell proliferation rates, even among follicles at the same stage of development. This parameter appears to influence whether a follicle can ovulate or become atretic.

## Teaser

Bromodeoxyuridine pulse-chase labeling revealed that only fast-growing follicles survived to ovulation and follicle growth modeling determined that initial follicle size or follicle growth rate can be advantageous for follicle survival.

## Graphical Abstract

**Graphical Abstract:**
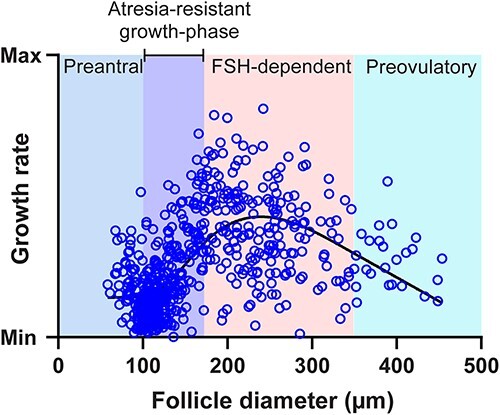

## Introduction

Ovarian follicles become increasingly sensitive to follicle-stimulating hormone (FSH) as they develop, eventually becoming entirely dependent on FSH stimulation for survival [1]. In mono-ovulatory species, selection of the ovulatory follicle begins with FSH-mediated “recruitment” of a cohort of developing FSH-dependent follicles. This occurs during the early part of the follicular phase and is known as the selection window because follicles that reach the FSH-dependent stage at other phases of the cycle will become atretic due to insufficient gonadotropin stimulation. As the recruited cohort develops, the output of estrogen and inhibin increases, suppressing pituitary gland FSH secretion via negative feedback. By the middle of the follicular phase, one “dominant” follicle emerges and grows toward the preovulatory stage, while the remaining follicles undergo atresia [2]. The dominant follicle survives by switching to luteinising hormone (LH) dependency, while the other follicles remain FSH dependent [3, 4]. The dominant follicle also produces large quantities of inhibin, to suppress FSH secretion (via negative feedback) and estrogen, and to stimulate LH secretion (via positive feedback) [5, 6]. The FSH suppression ensures atresia of all remaining antral follicles, while the LH secretion ensures survival of the dominant follicle [2].

The follicle destined to ovulate is presumed to have the highest FSH sensitivity in the cohort [7] and appears to be the first to attain LH dependency. The point where the subordinate follicles undergo atresia while the dominant follicle continues to grow is known as “deviation” and is well described in mono-ovulatory species via daily ovary ultrasound scan studies [8–12]. In many cases, the dominant follicle was not necessarily the largest follicle at the time of recruitment but exhibited the fastest growth rate [8, 10, 11], consistent with “race to LH dependency” concept. In polyovulatory species such as pigs, multiple preovulatory follicles accumulate in the follicular phase and the combined inhibin output of multiple follicles is apparently needed to effectively suppress FSH secretion [13, 14].

Mice ovulate around 10 oocytes per estrous cycle, which usually has a duration of 4–6 days (but longer cycle-lengths are not uncommon) [15]. The mouse follicular phase lasts the entirety of cycle and the corpora lutea persist across the subsequent three cycles [15–17]. The accumulation of preovulatory follicles during the cycle has been described in rats and mice but not with respect to the timing of atresia of the follicles that do not survive to ovulate [16, 17]. Based on the maximum sizes that follicles can reach when either FSH is absent, or when FSH is present but LH is absent, mouse antral follicles can be classed into three categories: FSH sensitive (130–200 μm diameter), FSH dependent (200–350 μm), and LH dependent/preovulatory (>350 μm) [4, 18–24]. The objectives of this study were to characterize the timing of mouse preovulatory follicle emergence and dominance (and concurrent atresia of the subordinate follicles) and to examine whether fast-growing follicles were more likely to ovulate than slow-growing follicles. Bromodeoxyuridine (BrdU) pulse-chase labeling was used to examine granulosa cell proliferation rates across follicle development and mathematical models were used to explore the effects of follicle growth rate on potential for selection into the preovulatory cohort.

## Methods

### Animals

Female C57Bl/6J mice aged 40–65 days were housed in individually ventilated cages with access to food and water ad libitum under 12 h light:12 h darkness cycles with constant room temperature (24 ± 1°C), with ad libitum access to standard rodent chow and water. Estrous cycles were determined by cytology of vaginal lavage fluid smeared on a glass slide, air dried, and stained with 1% w/v toluidine blue. The cycle stages were defined as follows: metestrus; high abundance of polymorphonuclear leukocytes with isolated nucleated epithelial cells, diestrus; proestrus; low abundance of polymorphonuclear leukocytes with isolated or clumped nucleated epithelial cells often accompanied by strand of mucous, proestrus; high abundance (>50%) of clumped, nucleated epithelial cells, low abundance of clumped, cornified epithelial cells, and absence of leukocytes or estrous; low abundance of nucleated epithelial cells, high abundance (>50%) of clumped cornified epithelial cells, and absence of leukocytes. Vaginal lavage was conducted daily for 7–21 days to determine the cycling patterns of each mouse. All animal experiment protocols were approved by the University of Otago Animal Ethics Committee.

### BrdU immunohistochemistry

Mouse ovaries were drop-fixed overnight in Bouin’s fixative and were wax embedded for serial sectioning at 5-μm section thickness. BrdU antigenic sites in the nuclear DNA were exposed in de-waxed slides by immersion in 1 mol/L hydrochloric acid at room temperature for 1 h followed by neutralization in 0.1 mol/L sodium borate buffer for 10 min. All antibody and blocking solutions were prepared in PBS with 0.2% Tween 20 and 0.1% bovine serum albumin. Blocking solution: donkey serum, Merck, cat# D9663, RRID: AB_2810235, diluted 1:20, 20-min incubation. Primary antibody: rat anti-BrdU, Abcam, cat#: ab6326, RRID: AB_305426, applied at 2 μg/mL, overnight incubation at 4°C. Secondary antibody: biotinylated donkey anti-rat IgG, Jackson Immuno Research, cat# 712-065-150, RRID: AB_2340646, applied at 3 μg/mL then streptavidin–biotin–HRP complex, Amersham, cat#: RPN1051, diluted 1:200, both incubated for 1 h at room temperature. Immunoreactivity was visualized with diaminobenzidine (DAB substrate kit, Vector Labs, cat# SK-4100) and the sections were counterstained with hematoxylin.

### Two-hour BrdU pulse-chase experiment

Mice were injected with a single intraperitoneal bolus injection of 50 mg/kg of BrdU (5-bromo-2′-deoxyuridine, Merck, cat# B5002) at 9:00 h at estrus, metestrus, or diestrus (*n* = 6 animals per timepoint). The animals were euthanized with anesthetic overdose (ketamine 3 mg/kg, medetomidine 225 mg/kg) 2 h post-injection. From each mouse, a series of 75 consecutive sections (375-μm-thick total volume) was chosen from one ovary for BrdU immunohistochemistry using a randomized start to select the first slide. Each follicle in the series was examined if the widest point of the follicle could be confirmed to be within the 375-μm-thick sampling volume. If the largest observable follicle diameter was in either for first or last section of the series, it was excluded because it was not possible to rule out the possibility that the widest point of the follicle was outside of the sampling volume. The cross-sectional area of the follicle at its largest size in the series was measured using ImageJ software [25]. The cross-sectional area was converted into a “pseudodiameter” by substituting the cross-sectional area into the formula for the area of circle and then calculating the radius of a circle with the equivalent area (Supplementary Figure S1). The pseudodiameter was used in place of cross-sectional area to enable comparison with the prior literature, where follicle size is most commonly described by diameter. Follicles were classified as atretic if they exhibited an accumulation of pyknotic nuclei in the antrum, a non-contiguous cumulus granulosa cell layer surrounding the oocyte, a pleated/wrinkled/rippled oocyte cell membrane, a pyknotic oocyte nucleus, or separation of the oocyte from the zona pellucida. Follicles were classified as non-atretic if none of these features were present.

### Forty-eight-hour BrdU pulse-chase experiment

Mice were injected with three intraperitoneal bolus injections of 100 mg/kg of BrdU during a single day, with injections at 10:00 , 12:00 , and 14:00 h. The mice were injected in three groups. Group 1 was injected on the first day of estrus and euthanized on the first day of metestrus. Group 2 was injected on the first day of metestrus and euthanized on the first day of estrus. Group 3 was injected on the first day of estrus and euthanized at proestrus. Serial sections were sampled at 125-μm intervals (every 25th section) and immunostained for BrdU with hematoxylin counterstain. Antral follicles were analyzed with the widest section in the series used to determine the pseudodiameter and the BrdU labeling index (LI).

### BrdU labeling index

The BrdU LI (proportion of BrdU-labeled granulosa cells) of the mural granulosa layer was calculated using pixel classification functions in the ilastik machine-learning software [26]. The software was trained to recognize and segment BrdU-labeled (BrdU^+^) and unlabeled (BrdU^−^) granulosa cells, antrum, oocytes, or areas of images containing no tissue. Cumulus granulosa cells were excluded from the LI measurements by manually cutting the cumulus region from the images prior to the pixel classification process. The segmented output was then imported into ImageJ where a region of interest was manually drawn around the basal lamina of the follicle and the LI was calculated using the following formula:


(1) \begin{equation*} LI=\frac{A_{BrdU+}}{A_{BrdU+}+{A}_{BrdU-}} \end{equation*}

where *LI* = labeling index, *A_BrdU+_* = area of BrdU^+^ granulosa cells, and *A_BrdU−_* = area of BrdU^−^ granulosa cells.

### Preovulatory follicle counts at proestrus

Serially sectioned ovaries were stained with hematoxylin and eosin as part of a prior study [27]. The pseudodiameter at the widest point was determined, as described above. All non-atretic follicles larger than 300 μm were counted (*n* = 6 mice).

### Mathematical modeling

Models were developed to determine how many days it would take a follicle of known diameter and LI to reach the preovulatory size. If the number of granulosa cells that will undergo cell division over a given time period is known, then the number of granulosa cells present can be calculated as


(2) \begin{equation*} {N}_i={N}_{i-1}+{N}_{i-1}. LI \end{equation*}

where *N* = the number of granulosa cells, *i* = an integer >0 representing the number of days since data were collected (i.e., data collected from animals euthanized 2 h after BrdU injection), and *LI* = BrdU LI. Cell division is often viewed as one “maternal” cell giving rise to two “daughter” cells, with the maternal cell no longer existing after division. This is an effective biological concept because newly synthesized, and original, maternal-cell DNA and organelles are shared between the two daughter cells and neither of which are identical to the maternal cell. However, mathematically it is possible to consider that after cell division, the maternal cell is retained, and one “budded-off” daughter cell splits of from the maternal cell. Thus, the number of original cells remains the same and the number of newly “budded-off” daughter cells increases by the number of cells that underwent cell division.

The growth formula can be iterated over multiple consecutive time periods. All iterations in the present study were performed over 24-h periods because ovulation can only occur at one time of day (the middle of the dark phase). Each new day represents a new opportunity to ovulate, if sufficient numbers of preovulatory follicles have accumulated over the last 24 h. However, the proliferation rate of granulosa cells declines as the follicle grows [16], hence multiple iterations require a correction factor (see Results) to reduce the size of the LI over time. This leads to the following modification of Equation (1):


(3) \begin{equation*} {N}_i={N}_{i-1}+{N}_{i-1}\ \left({LI}_0-f\left({N}_i-{N}_{i-1}\right)\right) \end{equation*}

where the subscript 0 indicates data obtained at day zero (i.e., data collected from animals euthanized 2 h after BrdU injection) and *f* is a correction factor obtained by multiplying the slope of a linear regression that describes decline in LI by the increase in follicle diameter since the first iteration and then multiplying by an adjustment factor, 0.795 (see Results). In the first iteration, where the follicle size increase is zero, the correction factor is set at 0.795.

With knowledge of the volume of a granulosa cell and the number of granulosa cells, it is possible to calculate the volume of all granulosa cells in the follicle. More specifically, the mural granulosa cell layer is the relevant structure for assessing follicle growth because proliferation of the cumulus cells is limited to a short distance around the oocyte [28] and therefore do not contribute to follicle growth. The mural volume shows an exponential relationship to follicle diameter (see Results) represented by the formula:


(4) \begin{equation*} {V}_M=0.18{D}^{3.05} \end{equation*}

where *D* = diameter of the follicle and *V_M_* = volume of the mural layer of the follicle.

Granulosa cell number can thus be converted into follicle diameter with the following formula:


(5) \begin{equation*} D=\frac{{\left(N.{V}_G\right)}^{\frac{1}{3.05}}}{0.18^{\frac{1}{3.05}}} \end{equation*}

where *V_G_* = volume of a single granulosa cell.

Combining Equations (3–5) generates the full iterative growth model.


(6) \begin{equation*} {D}_i=\frac{{\left(\left({N}_{i-1}+{N}_{i-1}\ \left({LI}_0-f\left({N}_i-{N}_{i-1}\right)\right)\right){V}_G\right)}^{\frac{1}{3.05}}}{0.18^{\frac{1}{3.05}}} \end{equation*}

### Statistical analysis

Linear and nonlinear regression curve-fitting was performed using Prism v9.5.1 (GraphPad software). Comparison of LIs between the different follicle size classes in the animals treated with BrdU followed by a 2- or 48-h chase period was conducted with a Kruskal–Wallis test in SPSS v28.0.1 (IBM) followed by Bonferroni-corrected pairwise comparisons as a post hoc test. Individual ovarian follicles were considered to be the experimental unit in all tests.

## Results

**Figure 1:**
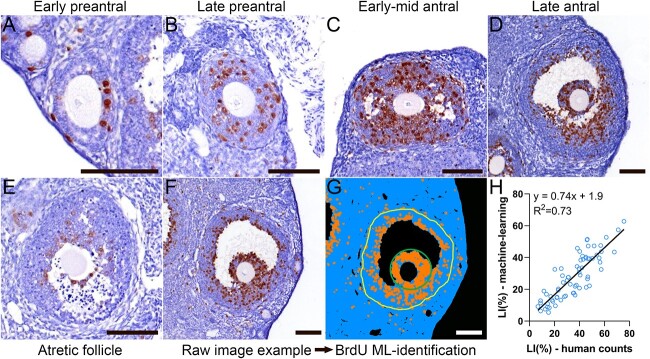
BrdU labeling in granulosa cells. BrdU was injected into female mice, followed by euthanasia 2 h later. BrdU in the ovaries was labeled by immunohistochemistry (brown stain) with hematoxylin counterstain (blue). Variable BrdU incorporation into the nucleus of granulosa cells was observed in early preantral (A), late preantral (B), early mid antral (C), late antral (D), and atretic (E) follicles. LIs for each follicle were generated by processing raw immunohistochemistry images (F) through machine-learning software (G) to identify BrdU+ cells (orange) and BrdU− (blue) granulosa cells within the granulosa layers of the follicle (yellow line shows location of the basal lamina, green line shows the area of cumulus cells removed from the LI determination). The number of pixels representing BrdU+ and BrdU− cells was used to generate a machine-learning-based LI which was compared to the LI generated by manual counting of BrdU+ and BrdU− cells (**H**). All scale bars = 100 μm. Abbreviation: ML, machine learning.

The mean (± SD) cycle length of the mice in the study was 6.2 ± 1.6 days, with a mean of 2.5 ± 1.3 days in estrus, 1.4 ± 0.6 days in metestrus, and 1.3 ± 0.6 days in diestrus. The proestrus stage reliably lasted for a single day in all animals. The proportion of BrdU^+^ granulosa cells was low in the early preantral follicles with successive increases during late preantral development and reaching a peak in the early antral phase (Figure 1A–C). The LI decreased with further antral follicle development (Figure 1D). Very little BrdU uptake was observed in atretic follicles, even in the histologically normal granulosa cells that were not yet showing overt signs of programmed cell death (Figure 1E).

Machine-learning pixel classification systems were evaluated as a high-throughput method to quantify the BrdU^+^ and BrdU^−^ granulosa cells. The segmented output accurately identified the labeled and non-labeled cells (Figure 1F and G). Comparison of the human-derived and machine-learning-derived LIs showed good agreement (Figure 1H), but the human estimates were 0.35-fold higher than the machine-learning estimates. This suggests that the human observer could better distinguish faint BrdU immunoreactivity, but the machine-learning method was high-throughput and more likely to be unbiased and reproducible. The machine-learning method was adopted for the study as the main objective was reliable comparison of proliferation rates between large numbers of follicles.

**Figure 2:**
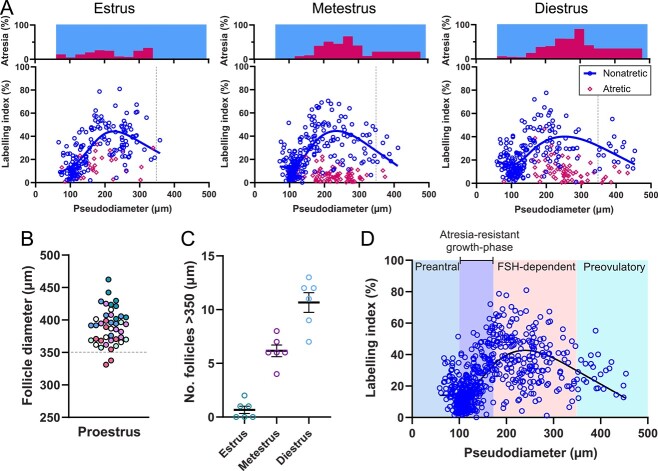
Granulosa cell proliferation rates in developing follicles. (A) Female mice were injected with BrdU either at estrus, metestrus, or diestrus and were euthanized 2 h later (*n* = 3 animals per timepoint). BrdU LIs for all follicles (non-atretic = blue circles, atretic = red diamonds) in the sampling volume were generated using machine-learning software and were plotted against the pseudodiameter of the follicle with a linear spline curve fitted to the data. Histograms above the scatter plots show the atresia rates in the corresponding follicle size classes on the *x*-axis (30-μm-diameter increments). The dashed gray line shows the putative size-threshold for preovulatory follicles (>350 μm). (B) Preovulatory follicle diameters were measured in a separate group of female mice at proestrus (*n* = 6, each symbol color represents follicles from an individual female). (C) The number of follicles with diameters >350 μm present in the ovaries of mice euthanized at estrus, metestrus, or diestrus (*n* = 6). (D) BrdU LIs for all follicles across all three stages of the estrous cycle (as shown in panel A) were combined into one growth trajectory plot categorized into four growth phases: preantral, atresia-resistant early antral, FSH dependent, and preovulatory (estrus *n* = 172, metestrus *n* = 246, diestrus *n* = 223, total *N* = 621 follicles).

Figure 2A shows the growth-rate trajectories of developing follicles by comparing LI to follicle diameter. Preantral follicles (80–130 μm diameter) had a slow rate of growth with LIs rarely above 30%. The follicles then underwent a “growth spurt” between the diameters of 130 and 200 μm, as evidenced by the large increases in the LI. Granulosa cell proliferation rates then showed steady declines as the follicles grew from 200 μm to full maturity. These growth patterns were similar at estrus, metestrus, and diestrus, albeit with the addition of larger antral follicles at each successive stage of the cycle. This suggests that the population of non-atretic follicles follows a relatively similar growth trajectory despite the differing levels of FSH secretion occurring at each stage of the cycle. However, there was substantial variability in the LI of follicles at the same size. It should also be noted that the total number of proliferating granulosa cells continues to increase as follicles grow, despite the decline in granulosa cell proliferation rates (Table 1).

**Table 1:** Average granulosa cell increase according to proliferation rate.

Follicle diameter (μm)	Mural layer volume	Mural granulosa cell count	Mean proliferation rate (%)	Cell number increase
100	2.3 × 10^5^	1.7 × 10^3^	49	8.2 × 10^2^
150	7.8 × 10^5^	5.7 × 10^3^	45	2.6 × 10^3^
200	1.9 × 10^6^	1.4 × 10^4^	41	5.6 × 10^3^
250	3.7 × 10^6^	2.7 × 10^4^	37	1.0 × 10^4^
300	6.5 × 10^6^	4.7 × 10^4^	33	1.6 × 10^4^
350	1.0 × 10^7^	7.6 × 10^4^	29	2.2 × 10^4^
400	1.6 × 10^7^	1.1 × 10^5^	25	2.9 × 10^4^
450	2.2 × 10^7^	1.6 × 10^5^	21	3.4 × 10^4^

Atretic follicles were rare at estrus, with increasing proportions of follicles at metestrus and diestrus, which are both known to be periods of FSH suppression. The onset of susceptibility to atresia occurred at a follicle diameter of ~170 μm, which is consistent with this being the beginning of the FSH-dependent phase of follicle development.

It was necessary to determine the minimum diameter of preovulatory follicles to examine the recruitment of the preovulatory cohort. The largest ovarian follicles on the morning of proestrus will ovulate in less than 24 h and thus represent preovulatory follicles, whereas medium antral follicles are largely lost due to atresia. The range of diameters of putative preovulatory follicles in mice euthanized at proestrus was 331–462 μm (Figure 2B). The preovulatory cut-off was set at 350 μm, as 95% of these large follicles were above this diameter. The number of follicles >350 μm was shown to progressively increase across estrus, metestrus, and diestrus, but sufficient numbers to represent a full cohort of 8–10 follicles first appeared at diestrus (Figure 2C). The rate of atresia in >350-μm-diameter follicles at diestrus (29%) was not sufficient to explain the complete removal and replacement of the >350-μm-diameter follicles present at metestrus. These findings suggest that the dominant follicle cohort is recruited sequentially, rather than arising as a single cohort. However, simply reaching the preovulatory size does not always guarantee follicle survival.

Combining the non-atretic follicles across estrus, metestrus, and diestrus reveals the average growth rate trajectory for the population of follicles, demonstrating the changes that occurs across the slow-growing preantral follicle stage (80–130 μm), the fast-growing atresia-resistant phase (130–170 μm), and the slowing growth rates in the FSH-dependent antral stage (170–350 μm) and the preovulatory follicle (>350 μm) phase (Figure 2D).

**Figure 3:**
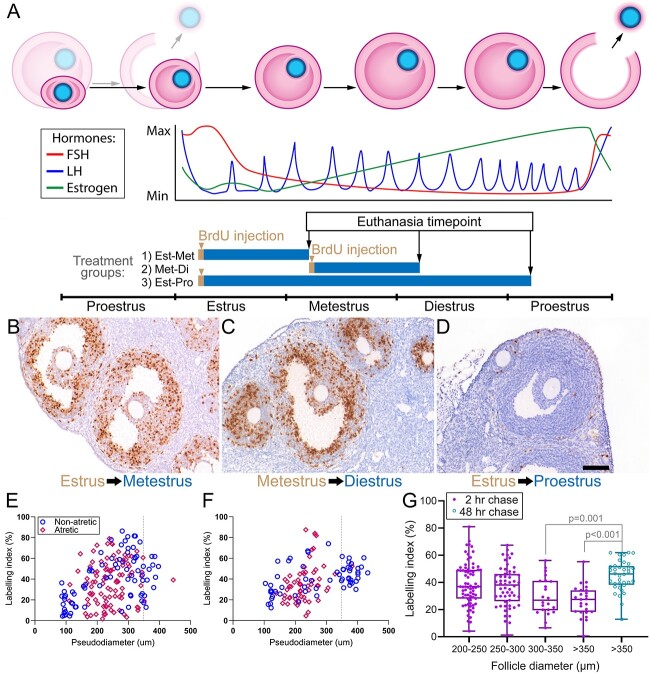
Long-term BrdU pulse-chase experiment. (A) Schematic of the BrdU injection and euthanasia regimen. The cycle begins with the ovulation of the preovulatory follicles in the preceding cycle at proestrus (half-shaded follicles) and ends with the development and ovulation of the next-largest cohort at the subsequent proestrus stage (full-shaded follicles). In group 1, the duration of estrus was 1 day in one mouse and 2 days in the other two mice. In group 2, the duration of metestrus was 1 day in one mouse and 2 days in the other two mice. Immunohistochemistry for BrdU in preovulatory-sized follicles in group 1 (B), group 2 (C), and group 3 (D), scale bar = 100 μm. BrdU LIs for follicles (non-atretic = blue circles, atretic = red diamonds) were plotted against the pseudodiameter for group 1 (E) and group 2 (F). (G) LIs of non-atretic follicles in the short term (2 h) were compared to non-atretic preovulatory-sized follicles in groups 1 and 2 in the long-term (24–48 h) pulse-chase experiment using Kruskal–Wallis test.

Long-term BrdU pulse-chase studies were performed to investigate whether growth rate affects the chances that a follicle will be selected to ovulate. In preliminary experiments, animals were injected with a single dose of BrdU on the first day of estrus and were euthanized at proestrus, but little or no BrdU immunoreactivity was observed in the preovulatory follicles (data not shown). This result suggested that the BrdU taken up by proliferating granulosa cells at estrus was being diluted out to an undetectable level by subsequent cell divisions. The dose of BrdU was therefore increased to three injections of BrdU over a 4-h period to increase the amount of BrdU incorporated into the dividing granulosa cells and shorter intervals between BrdU dosing and euthanasia were added (Figure 3A). Unfortunately, little BrdU immunoreactivity was observed in the granulosa cells animals injected at estrus and euthanized at proestrus but BrdU-labeled stromal and theca cells were observed indicating that BrdU uptake had occurred (Figure 3D). Fortunately, strong BrdU immunoreactivity was present in the granulosa cells of animals injected at estrus and euthanized at metestrus (Figure 3B), or injected at metestrus and euthanized at diestrus (Figure 3C).

The non-atretic LI versus diameter pattern in the estrus-metestrus group (Figure 3E) resembled the pattern at estrus (Figure 2A), albeit shifted to higher diameters. This confirmed that the long-term pulse-chase labeling reflected proliferation rates at the time of BrdU injection. If there had been loss of BrdU labeling due to high rates of subsequent granulosa proliferation, or if there had been preferential accumulation of BrdU^+^ daughter cells, the range of LI s would have changed. Interestingly, the LIs of atretic follicles in the estrus-metestrus group ranged from 2 to 79% indicating examples of both fast- and slow-growing follicles at estrus that had undergone atresia by metestrus.

In the mice BrdU-injected at metestrus and euthanized at diestrus, most of the follicles in the 170–350-μm-diameter range were atretic (Figure 3F). These atretic follicles tended to have LIs in the low range indicating that they had been slow growing at metestrus. In contrast, the preovulatory follicles (>350 μm) had LIs ranging from 13 to 62%, which is higher than the typical LI for preovulatory follicles (10–40%). If the preovulatory follicles at diestrus are assumed to have been in the 250–350 μm range at metestrus, then their LIs suggest that they were among some of the fastest-growing follicles in the cohort at the time of BrdU injection (compare Figure 3F to Figure 2D). The LIs of all preovulatory-sized follicles in the long-term pulse-chase study (from Figure 3E and F) were compared to LIs of all non-atretic follicles in the short-term pulse-chase study (from Figure 2D). This comparison (Figure 3G) showed that most preovulatory follicles would have had above-average growth rates in the 1–2 days prior to reaching 350 μm in diameter (*P* < 0.001, Kruskal–Wallis test). This is suggestive of a survivor effect caused by the preferential removal of the slow-growing follicles due to atresia.

**Figure 4:**
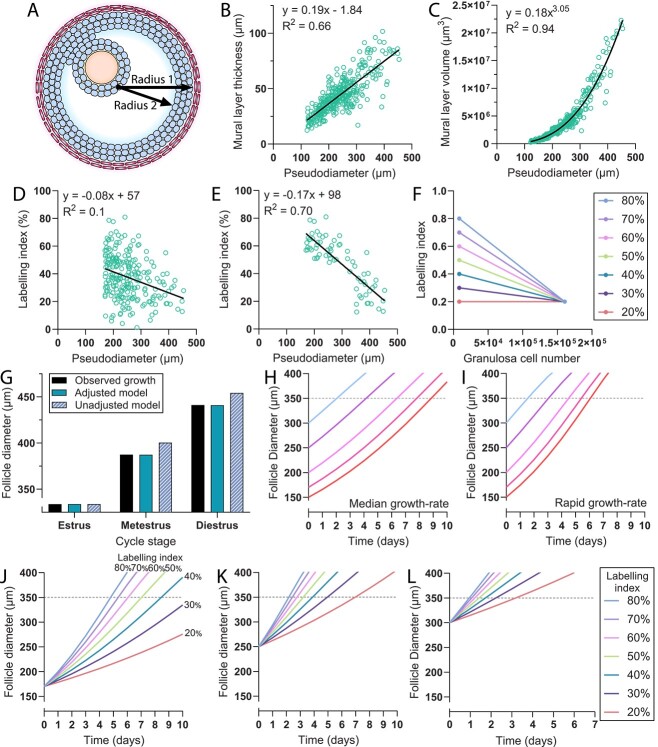
Modeling of antral follicle growth. (A) Follicle growth was calculated on the basis of how granulosa cell division would contribute to expansion of the mural volume, which can be calculated by formula for volume of a sphere of the whole follicle (radius 1) and then subtracting the volume of a sphere representing the antrum, including the cumulus-oocyte complex (radius 2). (B) The thickness of the mural layer exhibits a linear relationship with follicle diameter. (C) The mural layer volume exhibits an exponential relationship with follicle diameter. (D) Linear regression was used to characterize the “median” rate of change in BrdU LI as follicles increase in diameter (data were derived from all non-atretic follicles >170 μm in diameter from Figure 2D). (E) Linear regression used to characterize change in BrdU LI in fast-growing follicles was determined the top 20% of follicles in each 50-μm-diameter increment between 170 and 350 μm and all follicles >350 μm in Figure 2D. (F) Growth-related changes in BrdU LI set at 10% increments were also generated using hypothetical 170-μm follicles (representing the full range of observed LIs) and the average LI of preovulatory follicles. (G) The mean diameter of the five largest follicles at estrus, metestrus, and diestrus was used to represent the “observed” increase in follicle size, which was compared to follicle growth with the unadjusted model then subsequently an adjusted model (the final version of the model). Follicle growth over time was modeled in follicles of different sizes growing at the “median” growth rate (H) or the fast growth rate (I). Follicle growth was modeled in follicles of the same starting size and varied granulosa cell proliferation rate (J).

Mathematical modeling of follicle growth was conducted to explore how follicle growth rate could affect the potential for a follicle to enter into the dominant follicle cohort. The premise of the modeling was to determine the size of the mural layer at each follicle diameter (Figure 4A–C) and then the number of granulosa cells (132.6 μm^3^ per cell) required to fill the mural volume. Daily increases in granulosa cell numbers can be calculated from the BrdU LI, which increases the mural volume. The new mural volume then corresponds to a new, larger follicle diameter (Figure 4C).

Iterations of the formula were conducted to simulate growth across multiple days, but this required adjustment of the LI because granulosa cell proliferation rates are known to decrease with increasing development. Regression of LI versus diameter data from Figure 2D was used to generate rates of change in granulosa cell proliferation to represent follicles growing at median or fast speeds (Figure 4D–F) which was to be used in the correction factor calculation. To validate the model, the size increases of follicles during the estrous cycle were determined from the mean diameter of the largest five follicles at estrus (334 μm), metestrus (388 μm), and diestrus (441 μm). The use of raw BrdU LIs in the model led to follicle growth increases larger than these observations, suggesting that the model was overestimating granulosa proliferation rates (Figure 4G). To resolve this, the proliferation correction factor (*f*) was multiplied by 0.795, which led to close agreement between modeled and the observed follicle growth (Figure 4G). The growth of follicles with different granulosa cell proliferation rates was then modeled with this validated formula.

In the shortest possible estrous cycle (4 days), all preovulatory follicles would need to reach 350 μm in diameter within 3 days (the minimum time from estrus to diestrus). The modeling shows that it is not possible for a 250-μm follicle to do so when it is growing at a median rate but is possible if dividing at a rapid rate (Figure 4H). The modeling also shows that it is possible for a follicle to develop from the beginning of the FSH-dependent stage (170 μm) to preovulatory size (350 μm) within a single cycle, but only if the cycle is at least 5 days long (Figure 4J). The models suggest that if >10 follicles larger than 250 μm are present, particularly if growing faster than the average rate, then the cycle can be as short as 4 days (Figure 4K and L). However, if there are insufficient numbers of large follicles to fill the preovulatory follicle complement (or if too many undergo atresia before the preovulatory stage), smaller follicles can grow to eventually enter the preovulatory follicle cohort but the cycle will be lengthened.

## Discussion

The pattern of cellular proliferation in growing mouse follicles was very similar to that seen in historical studies in rats [16]. Follicles exhibit a slow-proliferating preantral stage followed by rapid growth in FSH-sensitive phase and then subsequent declines in granulosa cell proliferation thereafter. The phases match with the onset of FSH sensitivity and FSH dependency observed in vitro [23]. The use of long-term BrdU pulse-chase experiments demonstrated that the preovulatory follicles had previously been among the fastest-growing follicles in the FSH-dependent stage. Mathematical modeling of follicle growth rates appeared to confirm that follicles can develop from the beginning of the FSH-dependent stage (170 μm) to the preovulatory stage (350 μm) within the space of one estrous cycle, but only in cycles longer than 4 days. It is also likely that the follicle would have to develop at the fastest possible rate. Such a follicle appears capable of reaching the preovulatory size before a 250-μm follicle with a 30% LI (despite the 250-μm follicle having >3 times the number of granulosa cells at the start of the cycle).

The deviation of dominant follicles (the point where one follicle continues to grow alone while the others become atretic) is easily observed by ultrasound in mono-ovulatory species [8, 10]. Deviation is accompanied by strong suppression of FSH and increasing pulses of LH secretion [5, 6]. The analogous point in the mouse cycle appears to be diestrus, where the full complement of ~10 preovulatory-sized follicles arise, and most remaining antral follicles become atretic. This phase of the mouse estrous cycle also exhibits suppressed FSH secretion and rising LH secretion [29, 30].

The current theory is that polyovulatory species require more than one follicle to produce enough inhibin to suppress FSH secretion to the point that all other FSH-dependent follicles become atretic [14]. Within a single study, we have observed different C57Bl/6 mice ovulating 6 to 12 oocytes [31] indicating that variability can be high, even among genetically inbred females. In the present study, preovulatory follicles ranged from 350 to 450 μm in diameter, which corresponds to 7.6 × 10^4^ and 1.6 × 10^5^ mural granulosa cells, respectively. Based on total mural granulosa cell numbers, six 450-μm follicles can produce as much estradiol and inhibin as twelve 350 μm follicles, which could explain why it is possible for either 6 or 12 follicles to stimulate an LH surge. Other factors could be involved, particularly if thecal androgen production is a rate-limiting step (the above calculations apply to granulosa cells) or if hormone gene expression rates (e.g., inhibin genes) vary between follicles. However, in principle, differences in ovulation rates could be explained by variations in the size and number of preovulatory follicles in the dominant follicle cohort.

One of the key differences in mouse cycles is that there does not appear to be a clear selection window. The observation that there are some non-atretic 250–350-μm follicles present at estrus is curious because the FSH suppression at diestrus and early proestrus should have caused their demise on the prior cycle. A follicle cannot develop from the FSH-sensitive stage (170 μm) in the space of 1–2 days which suggests that large FSH-dependent antral follicles were capable of surviving a day or two with very low levels of FSH in the prior cycle. The 250–350-μm follicles have a clear advantage for reaching the preovulatory stage quickly, suggesting that surviving low FSH conditions at the end of the prior cycle is one strategy for surviving to ovulation.

A second strategy is for follicles to enter into the FSH-dependent phase in late proestrus and then grow rapidly in the hope that there are spare slots left in the preovulatory cohort. This argues against a clearly defined selection window in mice. However, while a fast-growing 170 μm at estrus would not reach the preovulatory follicle size within a 4-day cycle, it could reach 350 μm by metestrus of the following cycle. Furthermore, it would be in the 250–300 μm range at diestrus in the first cycle but would not have advanced to 300–350 μm range. In the present study, atresia rates were high in both size ranges, but non-atretic follicles were extremely rare in the 300–350 μm range and there might be a no-grow zone, specifically at diestrus. Therefore, follicles in the FSH-dependent phase may develop across two follicular phases in mice with 4-day cycles. This would also require sufficient numbers of atresia-resistant fast-growing ~170-μm follicles to fill a preovulatory cohort and they would all need to begin their growth at the same time, which is reminiscent of a selection window. However, this does not appear to be an essential requirement because the mouse ovary can seemingly compensate and recruit follicles sequentially over a longer cycle when required.

It is still not clear if variability in FSH sensitivity is simply random biological variation or if follicles can detect when they contain a high-quality oocyte and increase their FSH sensitivity accordingly. Oocytes secrete factors such as BMP15 and GDF9 that can increase granulosa cell proliferation and FSH [32, 33], but the genetic knockout of these factors leads to termination of folliculogenesis at the preantral stage [34, 35], which prevented investigation of the impact on the FSH-dependent stage. A recent study showed that ablation of the oocyte microvilli that project through the zona pellucida led to reduced GDF9 secretion and interestingly decreased granulosa cell proliferation [36]. Importantly, this effect was seen in follicles within the FSH-dependent size range. Therefore, there is a plausible link between oocyte communication and follicle development speed which in turn appears to influence the chances of follicle ovulation.

## Conclusion

This study confirms that fast growth rate in the FSH-dependent phase appears to be an essential characteristic of future preovulatory follicles. However, not all fast-growing follicles will be selected for ovulation, hence it also seems that a follicle must be in the right place at the right time, similar to follicles recruited in the selection window in mono-ovulatory species. Further research is needed to determine if there are additional features that make a follicle suitable for ovulation because the ultimate question remains: what is special about a developing follicle destined to ovulate, compared to its peers that are destined for atresia?

## Supplementary Material

Supplemental_Figure_S1_ioad134

## Data Availability

The data underlying this article will be shared on reasonable request to the corresponding author.
