# Supplementary material for: Ovarian follicle size or growth rate can both be determinants of ovulatory follicle selection in mice
Source: Biol Reprod. 2023 Oct 6;110(1):130–9. doi: 10.1093/biolre/ioad134 (PMC10790341; doi:10.1093/biolre/ioad134)
Supplement: Supplemental_Figure_S1_ioad134 [file supplemental_figure_s1_ioad134.pdf]

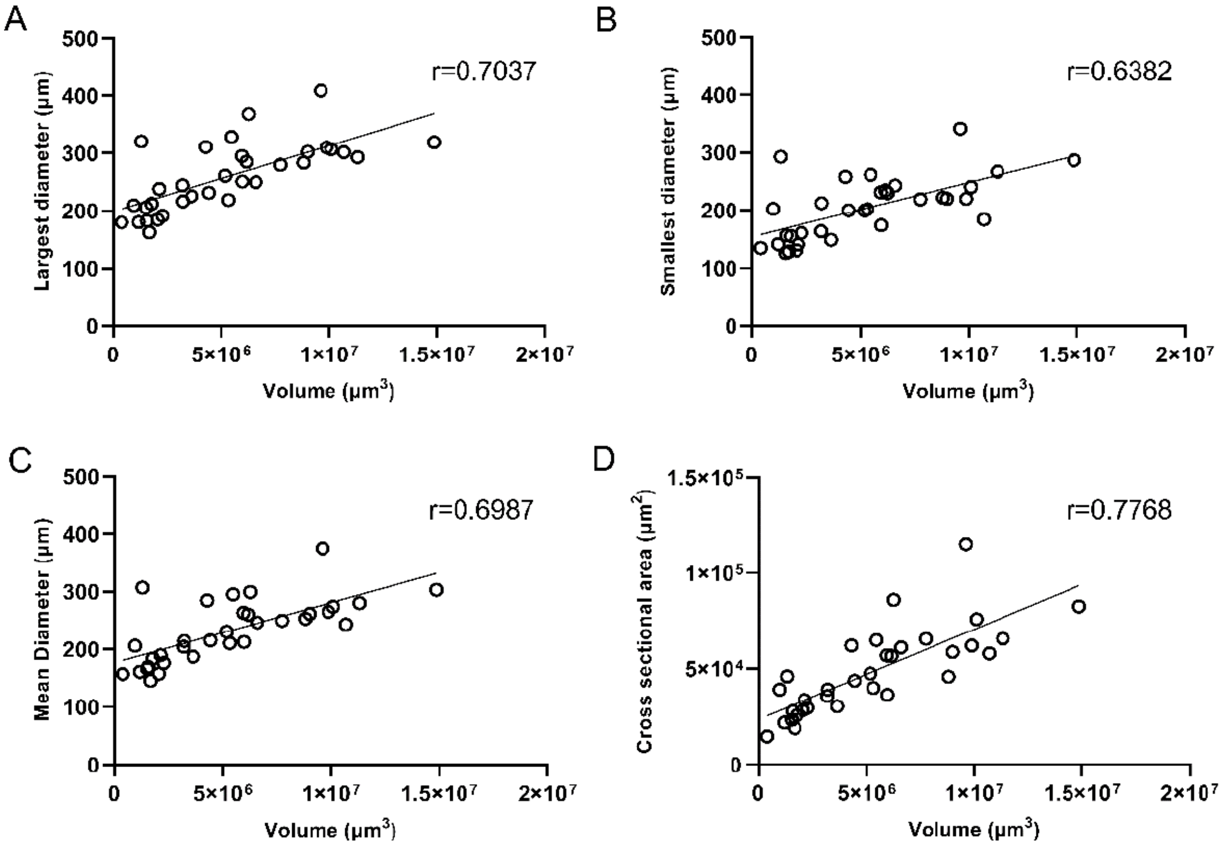

Supplemental Figure S1. Comparison of methods to quantify follicle volume from a single section. Total follicle volume was determined in a selection of follicles by multiplying the cross-sectional area of every 5th section in the follicle by distance between each sampled section (25 µm). The sum of these volume calculations for every section sampled throughout the follicle provided the total follicle volume (n = 34). In the largest cross-section in the centre of each follicle, the widest diameter (A), smallest diameter (B), mean of the largest and smallest diameter (C) and the cross-sectional area (D) were measured for linear regression against the total follicle volume. For all regressions $p < 0.001$. Cross-sectional area was found to have the strongest relationship to the total follicle volume and was chosen as the method to quantify ovarian follicle size.
